# Inhomogeneously distributed ferroptosis with a high peak-to-valley ratio may improve the antitumor immune response

**DOI:** 10.3389/fonc.2023.1178681

**Published:** 2023-08-28

**Authors:** Mau-Shin Chi, Der-Chi Tien, Kwan-Hwa Chi

**Affiliations:** ^1^ Department of Radiation Therapy & Oncology, Shin Kong Wu Ho-Su Memorial Hospital, Taipei, Taiwan; ^2^ Institute of Veterinary Clinical Science, School of Veterinary Medicine, National Taiwan University, Taipei, Taiwan; ^3^ School of Medicine, National Yang Ming Chiao Tung University, Taipei, Taiwan

**Keywords:** immunotherapy, ferroptosis, dose heterogeneity, modulated hyperthermia, nanoparticle, radiotherapy

## Abstract

Combined radiotherapy (RT) and mild hyperthermia have been used clinically for decades to increase local control. Both modalities tend to achieve a homogeneous dose distribution within treatment targets to induce immunogenic cell death. However, marked, and long-lasting abscopal effects have not usually been observed. We proposed a hypothesis to emphasize the importance of the peak-to-valley ratio of the dose distribution inside the tumor to induce immunogenic ferrroptosis in peak area while avoid nonimmunogenic ferroptosis in valley area. Although inhomogeneous distributed energy absorption has been noted in many anticancer medical fields, the idea of sedulously created dose inhomogeneity related to antitumor immunity has not been discussed. To scale up the peak-to-valley ratio, we proposed possible implications by the combination of nanoparticles (NP) with conventional RT or hyperthermia, or the use of a high modulation depth of extremely low frequency hyperthermia or high resolution spatially fractionated radiotherapy (SFRT) to enhance the antitumor immune reactions.

## 1 Introduction

Radiotherapy (RT) is among the most well-known immunogenic cell death (ICD) inducers, especially stereotactic ablative RT (1), which stimulates adaptive immunity by releasing damage-associated molecular patterns (DAMPs) (2). ICD and dendritic cell (DC) maturation are the two most crucial elements for successful *in situ* vaccination by RT. Ferroptosis is an iron-dependent ICD caused by the accumulation of oxidized phospholipids, leading to membrane damage and the release of immune-stimulatory cytokines, chemokines, and danger signals to elicit an immune response (3). Ferroptosis occurs in many different treatments, such as chemotherapy, targeted therapy, RT, and hyperthermia (4, 5). Besides direct DNA damage, RT activates massive reactive oxygen species (ROS) production, which may cause ferroptosis (6). The cell death from hyperthermia by radiofrequency ablation, nanoparticle-based hyperthermia, magnetic field hyperthermia or photothermia all closely related with toxic ROS production (7–9). The observation of ferroptotic death depends on the defense mechanism of cells such as the amount of glutathione peroxidase 4 to detoxify lipid peroxides, and the treatment dose intensity of producing ROS (10). While ferroptosis may play a role in immune stimulation, its potential negative effects (non-immunogenic ferroptosis) in treatment-induced progression are recently noticed. For example, tumor associated neutrophils died by ferroptosis are associated with immune suppression (11). Excessive oxidized lipids from ferroptosis can also have negative consequences in CD8+ tumor infiltrating lymphocyte (12). In the three-dimensional (3D) *in vivo* situation, the DAMPs and cytokines released from tumor after treatment (including but not limited to RT) may have different actions on immune response depending on the context of cancers.

There are many tumor-associated cells in the 3D tumor environment, and many of them are immunosuppressive. How can we induce cancer specific ferroptosis from cancer cells while avoid non-immunogenic ferroptosis from tumor infiltrating immune cells after treatment? The simplistic way is to create an inhomogeneous dose distribution inside tumor, in which a small area of ablative high dose deposit simultaneously within larger area of non-toxic low dose. The ablative dose results in an abundant accumulation of sufficient lipid peroxides to trigger a robust ferroptosis, while sparing most non-cancer tumor infiltrating immune cell death in the low dose area. In minibeam proton therapy (MPT), the dose profiles are areas of high dose (peaks) separated in between areas of low dose (valleys). MPT delivered as an array of quasi-parallel microbeams with tens of microns and spaced by hundreds of microns to generate a peak-to-valley ratio of more than 10 (13). The center-to-center distance between microbeams can be 5-10 times wider than that of minibeam. The high peak-to-valley ratio significantly increased the therapeutic index and strong anti-tumor immune response in rat glioma model by MPT than standard proton therapy (14, 15). The term peak-to-valley ratio originates from spatially fractionated RT, which is characterized by multiple hot and cold dose subregions within the treatment volume. It is defined as the ratio of the highest physical radiation dose to the lowest dose. In this article, the term was used to specify the idea of an inhomogeneous dose distribution, where the high dose intensities have reached the lethal threshold of ROS associated iron-dependent ferroptosis. Unlike the physical dose measurement, the peak-to-valley ratio is a conceptual parameter when describing the drug distributions from blood vessels to tumor tissues or the thermal dose distribution during hyperthermia treatment.

Despite the long history of combined conventional RT, chemotherapy and hyperthermia treatment, dramatic and long-lasting abscopal effects were not usually seen. We are interested in exploring why the relatively new techniques of RT, spatially fractionated radiotherapy (SFRT), and one of the hyperthermia techniques, amplitude-modulated hyperthermia (mHT), have reported more abscopal effects than conventional RT or hyperthermia (16–18). Many studies have shown that the nanoparticles (NP) can induce ferroptosis and immunomodulation (19). Taken together, we cocategories the immunogenic, non-immunogenic ferroptosis, SFRT, MPT, mHT, NP and immune modulation effect into the following hypothesis.

## 2 The hypothesis

**Figure 1:**
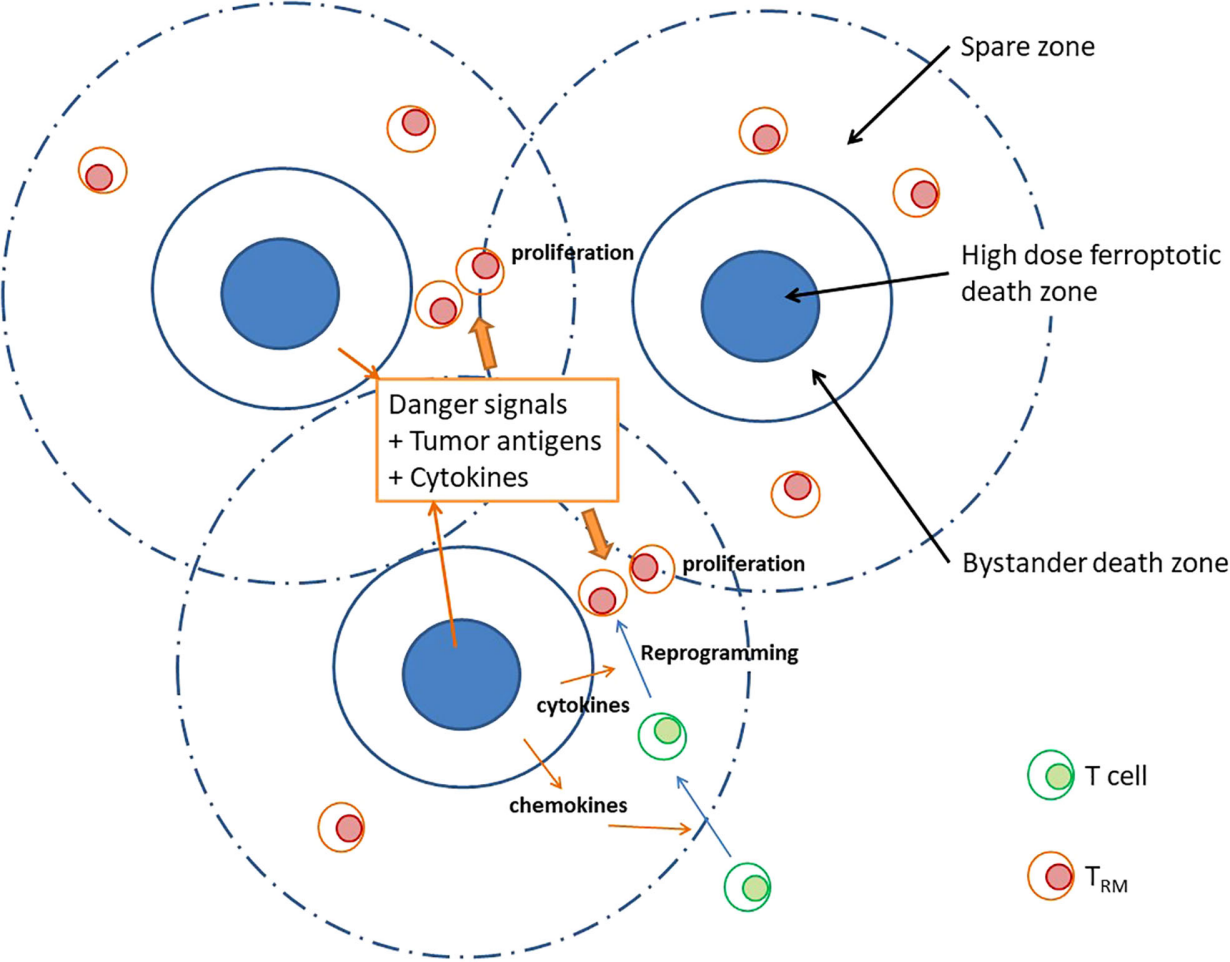
A high peak-valley ratio may provide free spaces for effective immunological chain reactions besides ferroptosis occurs in high peak area. In a treatment characterized by high peak-to-valley ratio, we may observe an inhomogeneously distributed “high dose ferroptosis death zone” within high dose area. These ferroptotic signals effectively propagate to the surrounding regions, thereby ensuring bystander killings. In addition, secretion of danger signals, tumor antigens, and cytokines plays a crucial role in reprogramming tissue resident memory T cells (TRM) within the “spare zone.” This cascading effect serves a dual purpose by not only aiming to enhance the therapeutic index but stimulates an immune response.

Ferroptosis is observed clustering in nano, tiny-, or small areas in the tumor. Although the high dose volumes are relatively small, the contact volumes between high and low doses are significantly large. Inhomogeneously distributed ferroptosis occurred in the peak dose areas within tumors prevent the production of non-immunogenic ferroptosis from their surrounding tumor infiltrating cells. The ferroptotic signals emanating from the peak dose areas can propagate to the immediate surrounding areas to ensure bystander killing and extending the biological consequences to a further distance. The secreted danger signals, chemokines, tumor antigens, and cytokines in the post-treatment tumor microenvironment (TME) may help reprogramming of newly recruited T cells reside in the “spare zone”. As shown in **Figure 1**, the tissue resident memory T cells (TRM), the key anti-tumor T cells, may therefore increase through a less damaged, better adapted and newly recruited way. This high peak-to-valley ratio of ferroptosis within tumors not only aims to increase the therapeutic index but has the potential to boost the immune response.

## 3 A large contact surface area between ferroptotic cells and spare space cells may avoid over-inflammation

Ferroptosis is characterized by the accumulation of iron-dependent ROS, with the property of propagating to neighboring cells along with lipid peroxidation and iron with a time-dependent random surges, oscillations, and chemokines gradient propagation (20). Riegman et al. quantified the nonrandom patterns of cell death through live-cell imaging. Only iron-dependent ferroptosis exhibited a wave-like spreading pattern of cell death but not necroptosis or apoptosis (21). Lipid peroxidation, leads to conformational changes (ferroptic pores) on the plasma membrane with death-inducing component leakage. In ferroptosis, the phenomenon of bystander cells killing differs from what is observed in the radiation-induced bystander effect (RIBE) (22, 23). RIBE can increase the proportion of cell death, whereas tsunami-like ferroptotic death leads to massive cell eradication (21). In the case of immune activation, a staggered pattern of DAMPs may be more efficient than a constant or uniform level because it allows for a graded response for better homeostasis response. Ferroptosis in tumor is a double-edged sword in which a massive number of DAMPs may be fulminate, while a relatively low concentration of DAMPs may help to promote tissue repair and regeneration, without inducing excessive inflammation (24). A decreased Immunoscore in the TME usually represents a sign of clinical improvement after treatment (25). A persistent inflamed TME after treatment usually indicates over-inflammatory. Resolvins are potent endogenous anti-inflammatory mediators derived from omega-3 polyunsaturated fatty acids (PUFAs) (26). PUFAs are the central components for ferroptosis; interestingly, PUFAs are also key components involved in regulating the resolution of inflammation (27). For metabolites of lipids to initiate and resolve inflammation, both must be produced in sufficient amounts in the right locations with right proportions.

## 4 A larger proportion of TRM cell can survive clinically with higher peak-to-valley ratio treatment

Arina et al. had reported that instead of been damaged by RT, a large proportion of preexisting tumor TRM CD8 T cells survive and mediate antitumor responses (28). In multi-cancer types, the abundance of TRM phenotype in tumor correlates with longer disease-free and overall survival (29, 30). TRM cells are characterized by the expression of CD103 and CD69, which are more radio-resistant than circulating/lymphoid tissue T cells may survive in irradiated tumor. They can produce higher interferon gamma after irradiation and kill the cancer cells (31). TME comprises diverse cells with distinct secreted molecules. Untreated cancer and stromal cells can suppress the activity of TRM cells by releasing immunosuppressive molecules as well as the recruitment of immunosuppressive tumor infiltrating cells. En bloc eradication of tumors with high-dose radiation not only demises the cancer cells and immune suppressive cells, but also results in a reduction in the absolute quantity of TRM. While high peak to valley ratio treatment can selectively increase the proportion of TRM because of their relative resistance to treatment. The low dose regions help to spare cell damages and provide a “reserve space” that peripheral T cells can migrate into tumor. The danger signals, Th1 cytokines, and the chemokines from ferroptotic cells can help the reprogramming of newly infiltrating T cells to TRM cells.

## 5 Three clinical implementations that can validate the hypothesis

### 5.1 SFRT might trigger antitumor immunity more efficiently

SFRT, recently called lattice RT, has used clinically for managing large tumors while limiting toxicity to adjacent normal tissues by delivering inhomogeneous high doses of radiation to different areas within the gross tumor volumes by conventional RT. It is typically irradiated with only a few small dot-like volumes in a bulky tumor with a high per fraction dose (15-20 Gy) while constraining the peripheral target dose within the tolerance limit (32, 33). The center-to-center distance (range 2-6cm) and vertices diameter (range 0.8-1.5cm) and the peak-to- valley ratio are not as impressive as MPT (34). Whether inhomogeneous dose planning is better than homogeneous dose planning remains a matter of clinical debate. Lucia et al. reported that an inhomogeneous tumor dose distribution provided better local control than a homogeneous distribution in stereotactic RT for brain metastases, wherein a dose of 3 × 7.7 Gy was administered in the periphery and that of 3 × 11 Gy at the isocenter (35). The 1-year local control rate was 78% versus 93% (p = 0.005) in favor of an inhomogeneous dose distribution (35). The current understanding of the biological effect of SFRT is mainly based on the bystander effect in which significant killing is observed next to the high dose radiated region and endothelial damage in the tumor microenvironment (36, 37). The effect of SFRT on the immune response has been reported. Kanagavelu et al. reported that under the same maximum dose of 20 Gy covering 20% of tumor volume led to maximum growth delay of distant tumors (abscopal effect) compared 20 Gy of RT to whole tumor volume (38). A more robust interferon-gamma response was noted in partial volume-irradiated tumors compared to whole-tumor irradiation (38). When the radiation dose is distributed heterogeneously, some regions of the tumor may receive an exceptionally higher dose of radiation than others. The regions that receive the highest dose of radiation may experience a greater degree of ferroptosis and a more pronounced release of tumor associated antigens (TAAs) and DAMPs, leading to a stronger immune response. In contrast, whereas the radiation dose is distributed homogeneously throughout the tumor, each region receives a moderate dose of radiation to avoid toxicities. This may result in a relatively weaker immune response as the release of TAAs and DAMPs are modest.

### 5.2 Low-frequency radiofrequency may produce an inhomogeneous heating and abscopal effect

The low-frequency RF energy was thought to be absorbed on the raft area of the cell membrane (39). Extremely low frequency RF-generated electric field (50 Hz) has been proven to increase serum lipid peroxide substances and superoxide dismutase activity from *in vivo* experiments (40). The mEHT (EHT2000, Oncotherm Kft., Hungary) is a widely applied hyperthermia technique that involves the simultaneous delivery of an appropriate low frequency (100 Hz to KHz) to modulate the 13.56-MHz carrier frequency. The fundamental concept of this machine is to specifically target the low-frequency energy carried by high-energy RF hyperthermia. Although the low-frequency RF has limited penetration capability, ithas a higher biological influence (39). As a result, mEHT selectively produced inhomogeneous heating specifically on the membrane raft area, and the apoptosis effect of mEHT treatment compared to water bath, using different reference calibrations, was shown to be approximately 4°C above (41). High intensity RF induced heat shock may cause ICD (42). mEHT was reported to have some abscopal effects observed (18) and to improve the result of intratumoral DC immunotherapy (43). The absorption of low frequency RF waves in biological tissues is primarily due to the interaction of the electromagnetic field (EMF) with charged particles (e.g., ions and electrons) in the tissue. When an RF wave passes through a tissue, the charged particles in the tissue are set into motion, which results in energy transfer from the EMF to the tissue. At the junction of fractal division, which refers to the point where a branching pattern of blood vessels or other biological structures splits into smaller branches, the tissue is particularly susceptible to absorption of RF energy (44). This is because the junction of fractal division typically has a higher density of charged particles compared to other areas of the tissue, which allows for more efficient energy transfer from the RF wave to the tissue.

### 5.3 NP-based ferroptosis frequently triggers antitumor immunity

The advantages of various biomaterial-constructed NPs were characterized by better biocompatibility, cytotoxicity and targeted delivery. The dispersion of NPs inside tumors is by nature heterogeneous and appears to be clustered throughout the organism. Drastic energy absorbed to the NPs at membrane, cytoplasm, or mitochondria on the nanometer scales suggests hundreds to thousands fold stronger dose needed over the centimeter scales. Both iron-based and noniron-based NPs may selectively accumulate in tumors, increase free iron, oxidized lipids, ROS, ferroptosis and are frequently reported to augment antitumor immunity (45–47). Ferroptosis induced by iron-containing NPs has gained more attention because of its imaging applicability and the synergism of combined NPs with magnetic fields, RT, laser irradiation and RF treatment (48–51). Macrophages are a type of immune cell that may engulf and digest cell debris, including NPs. Tumor-associated macrophages, the so-called immune-suppressive protumor-type macrophages, are prone to undergoing ferroptosis (52). Therefore, iron-containing NPs can selectively inhibit tumor growth by polarizing proinflammatory macrophages around the tumor (53).

## 6 Conclusions

ICD through ferroptosis is widely applied to trigger antitumor immunity. Many traditional ferroptosis inducers, such as conventional RT, drugs and hyperthermia, cannot generate clinically significant immune responses. Nevertheless, newly developed ferroptosis inducers, such as various NPs, SFRT, MPT, and low frequency RF hyperthermia, are all characterized by randomly or selectively inhomogeneous ferroptosis production within tumors. Our hypothesis suggests that inhomogeneous dose distribution is not a disadvantageous issue; in contrast, the high peak-to-valley ferroptosis within tumors would be a viable strategy to modulate TRM for cancer therapy. Based on this hypothesis, the synergistic use of NP can sensitize conventional RT or low frequency RF hyperthermia treatment, thereby rendering the tumor microenvironment more immunogenic. Such strategy holds a potential for future clinical translation.

## Data availability statement

The original contributions presented in the study are included in the article/supplementary material. Further inquiries can be directed to the corresponding author.

## Author contributions

K-HC, D-CT contributed to conception and design of the study. M-SC wrote the first draft of the manuscript. K-HC wrote sections of the manuscript. All authors contributed to manuscript revision, read, and approved the submitted version.
